# Clinical outcomes and radiolucent line analysis in cementless mobile-bearing total knee arthroplasty: a prospective multicentre study in Japan

**DOI:** 10.1038/s41598-024-71806-4

**Published:** 2024-09-08

**Authors:** Yukihide Minoda, Shigeru Nakagawa, Hideki Ueyama, Hideki Warashina, Michitaka Kato, Tomoyuki Matsumoto, Masahiro Nozaki, Makoto Kobayashi, Yukie Horikoshi, Junko Yasuda

**Affiliations:** 1 Department of Orthopedic Surgery, Osaka Metropolitan University Graduate School of Medicine, 1-4-3 Asahimachi, Abeno-ku, Osaka, 545-8585 Japan; 2 Osaka Rosai Hospital, Osaka, Japan; 3 Nagoya Orthopaedic Clinic, Nagoya, Japan; 4 Kobe University Graduate School of Medicine, Kobe, Japan; 5 Nagoya City University Medical School, Nagoya, Japan; 6 Johnson & Johnson K.K. Medical Company, Tokyo, Japan

**Keywords:** Diseases, Rheumatic diseases, Osteoarthritis

## Abstract

The objective of this study was to assess radiolucent lines (RLLs) and to determine their effect on clinical outcomes of the newly introduced cementless mobile-bearing total knee arthroplasty (TKA) system. This was prospective, multicentre study. Seventy-eight patients with knee osteoarthritis who underwent primary TKA were enrolled. Patient-reported outcome measures (PROMs) and radiographic assessments were evaluated at preoperative baseline and at 6 weeks, 1 year, and 2 years after surgery. KOOS, PKIP, 2011KSS, EQ-5D-3L and SKO improved from preoperative baseline to all postoperative timepoints, with no loosening of components. No RLLs were detected at 6 weeks after surgery. However, RLLs ≥ 1 mm developed in 2.8% of the patients for the femur and 9.7% for the tibia at 1 year after surgery, and values were 5.7% and 10.9%, respectively, at 2 years after surgery. RLL incidence was not correlated with PROMs. Age, sex, body mass index, range of motion knee flexion, posterior cruciate ligament treatment and β angle did not impact the occurrence of RLLs. There were no intraoperative complications, revisions or reoperations. This TKA system improved PROMs and showed less incidence of RLLs compared to the previous reported TKA without implant-related complications.

## Introduction

Despite the high prevalence and surgical success of total knee arthroplasty (TKA), debates regarding the optimal fixation method, cemented versus cementless, remain unresolved. TKA represents a widely performed elective surgical intervention to alleviate pain, most frequently associated with osteoarthritis. Ageing is a risk factor for osteoarthritis, and the number of patients in Japan undergoing TKA is expected to increase over the next 10 years in most age groups, indicating a high demand for primary TKA^1^. More than 80% of TKAs in Japan involve cemented fixation, indicating that this approach remains the gold standard^2^. However, there has been a recent increase in the demand for cementless fixation, which involves osteointegration between an implant and bone^3^. Long-term fixation is one of the challenges in TKA, and aseptic loosening is the leading reason of revision^3^. Hence, finding alternative anchoring techniques is becoming increasingly crucial. The design of cementless knee prostheses has evolved over the last 30 years. Introduction of new materials as well as coating such as tantalum, hydroxyapatite coating, porous metals and 3D printing improved biological fixation of implants. Keels and longer multiple pegs were added to support better survivorship of the cementless TKA^4,5^. Long-term follow-up studies have confirmed that cementless TKA achieves good initial fixation that is sufficient to allow good bone ingrowth and deliver reliable long-term fixation^6–9^.

Although the fixation of cementless TKA is improving, some surgeons hesitate to use cementless fixation in elderly patients owing to concerns regarding successful bone biological fixation. Early studies of various cementless tibial components showed a high incidence of radiolucent lines (RLLs)^10,11^. While newer cementless knee implants have potentially improved upon various aspects, RLLs continue to be a concern. Several reports of cementless TKA and the impact of RLLs on patient outcomes and risk factors, such as age and body mass index (BMI), have been published^12,13^. The literature review, such as those of Salem et al.^14^ describes the clinical outcomes of studies with several patient characteristics, however, the studies comprehensively evaluated the risk factors, clinical outcomes, and RLLs are still few.

A newly introduced cementless mobile-bearing TKA system (Attune^®^; DePuy Synthes, Inc., Warsaw, IN, USA) was designed to provide advanced optimal fixation compared with previous designs. A former cementless system (LCS; DePuy Synthes, Inc., Warsaw, IN, USA) is a clinically successful implant with a survival rate at 10 years of 98.9% (95% confidence interval [CI] 92.1–99.8)^15^. Design of the newly introduced system used the knowledge from previous implant designs, including the LCS. The tibial baseplate of the newly introduced system was designed with four pegs positioned radially from the central cone of the tibial base. Porocoat^®^ porous coating (DePuy Synthes, Inc.) was applied to the underside of the tibial baseplate, fully coating the four pegs, and was also applied on the proximal surface of the central cone. The placement of the Porocoat^®^ porous coating was designed to reduce micromotion, thus encouraging biologic fixation.

This prospective multicentre study involved 4 sites in Japan, specifically aims to investigate the incidence and progression of RLLs after cementless TKA and to determine their effect on clinical outcomes of the newly introduced cementless mobile-bearing TKA system (Attune^®^) in the early clinical stage. The relationship between possible risk factors and the incidence of RLLs 2 years after surgery in patients who underwent primary TKA with the cementless mobile-bearing system was also investigated. The study hypothesis was that the incidence and progression of RLLs with the study implant (Attune^®^) are lower than those in previous reports of patients who received an existing cementless TKA implant (LCS)^15^, and that RLL progression affects the clinical outcomes. The study result enables to understand a comprehensive short-term performance of the newly introduced system in the Japanese patients.

## Methods

This was prospective, multicentre clinical case series study (NCT03193034). The study design was selected to confirm the short-term performance of the implant during clinical use in the Japanese patients at a limited number of sites specialized in TKA, before introducing this implant widely in Japan. The study was approved by multiple ethics committees of the participating institutions (Kobe University [290010], Osaka Rosai Hospital [29-17], Nagoya City University [60-17-0043], Nagoya Orthopaedic Clinic [E2017-04-001]) ensuring complied with the Declaration of Helsinki and the Ethical Guidelines for Medical and Health Research Involving Human Patients (Public Notice of the Ministry of Education, Culture, Sports, Science and Technology and the Ministry of Health, Labour and Welfare No. 3 of 2014; partially revised on 28 February 2017). Written informed consent was obtained from each patient participating in this study.

Eighty-one patients who underwent primary TKA from July 2017 to October 2018 were evaluated. The inclusion criteria were as follows: (1) aged between 22 and 80 years at the time of surgery, (2) diagnosed with non-inflammatory degenerative joint disease resulting from osteoarthritis or post-traumatic arthritis, and (3) not bedridden. The exclusion criteria were as follows: (1) diagnosed with inflammatory arthritis, (2) diagnosed with radicular pain originating from the spine, and (3) with a muscular disorder that limits mobility. The Attune^®^ cruciate-retaining rotating platform cementless total knee system was used in all included patients. Surgery was performed using standard instruments, in accordance with the manufacturer’s instructions. For distal femoral bone cut, intramedullary rod was used. For proximal tibial bone cut, extramedullary rod was used. Patellar resurfacing and non-resurfacing were determined on the basis of the surgeon’s preference. Patients were allowed to walk the day after surgery.

Clinical evaluations were assessed using the following patient-reported outcome measures (PROMs): Knee Injury and Osteoarthritis Outcomes Score (KOOS), Patient’s Knee Implant Performance (PKIP) Questionnaire score, 2011 Knee Society Score (2011KSS) and European Quality of Life 5 Dimensions—3 Levels score (EQ-5D-3L), as well as Subject Knee Outcomes (SKO) scores from preoperative baseline to 6 weeks, 1 year, and 2 years after surgery. To collect various conditions and symptoms of the patients, multiple PROMs were collected. These evaluations contain sub-scores that encompass symptoms, routine functional activities to more advanced activities, which is a relevant manner for assessing recovery.

**Fig. 1:**
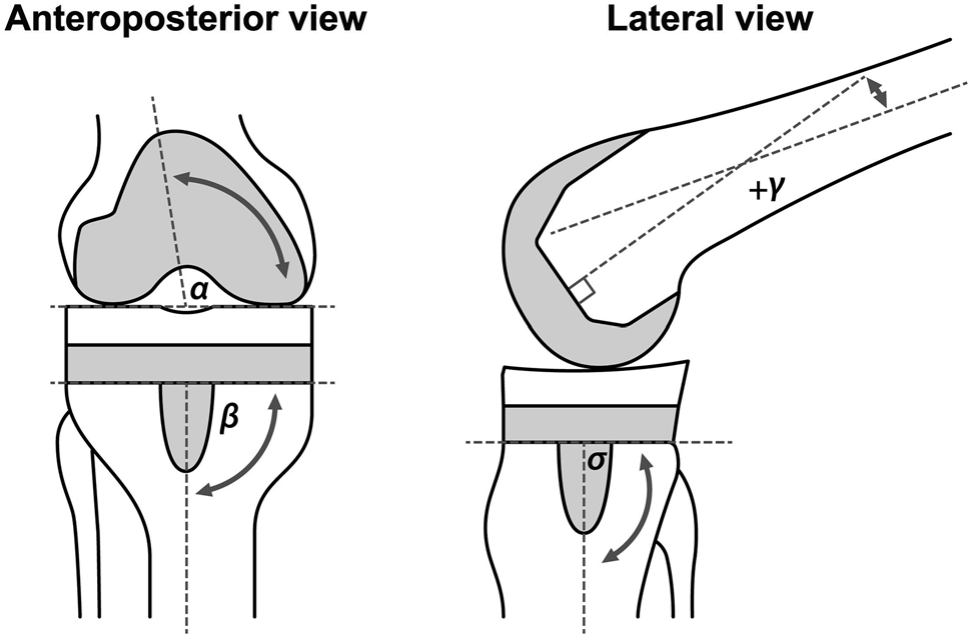
Radiographic evaluation of the implanted femoral and tibial components. The distal femoral valgus angle (α) and proximal tibial varus angle (β) were assessed on anteroposterior radiographs, and the femoral flexion angle (γ) and tibial slope (σ) were assessed on lateral radiographs to evaluate the alignment of the components.

To evaluate alignment and RLLs, the radiographic unilateral weight-bearing anteroposterior view and lateral view were obtained at preoperative baseline and at 6 weeks, 1 year, and 2 years after surgery. The preoperative and postoperative femorotibial angle (FTA), postoperative implant alignment according to the criteria of the Knee Society^16^ (Fig. 1) and fixation of the implant through zonal radiographic analysis of the RLLs were measured at 6 weeks, 1 year, and 2 years after surgery. The KOOS and 2011KSS subscores by the RLL width at 2 years after surgery were also evaluated. All radiographs were evaluated by an independent trained external expert radiographic reviewer at a contracted outside venue. The reviewer was a board-certified, fellowship-trained, licensed, and practicing musculoskeletal radiologist with no financial interest in sponsored company of this study.

All the potential risk factors for the development of an RLL at 2 years after surgery that collected during the study were included in the models for uni- and multivariate analyses: age, sex, BMI, range of motion (flexion), posterior cruciate ligament treatment, FTA and proximal tibial varus (β) angle.

Intraoperative adverse events were evaluated until the end of the study. Survivorship of the implant was assessed at 2 years after surgery.

### Statistical analysis

Referring to previous internal study data, the change in the KOOS-Activities of Daily Living score from preoperative baseline to 1 year after surgery was anticipated to be a mean of 32 points and a standard deviation (SD) of approximately 19 points. A sample size of 68 patients was likely to result in a 95% CI with a margin of error of ± 4.52. Considering a loss to follow-up of up to 15%, a sample size of 80 patients was planned.

Descriptive summaries for continuous data comprised mean and SD values. For categorical data, the number and percentage were provided. Both descriptive summaries and categorical data were based on the number of patients without missing data. Only actual subject data collected was included in final analyses. No imputation of missing data was performed. Two-sided 95% CIs using the t-distribution/binomial exact method were presented for continuous and categorical data. The paired t-test was used for comparisons between each postoperative timepoint and preoperative baseline. Uni- and multivariate logistic regression models were used to estimate the unadjusted and adjusted odds ratios, respectively.

A two-sided alpha of 0.05 was used for statistical testing. All statistical analyses were performed using SAS version 9.4 (SAS Institute, Inc., Cary, NC, USA). The following section presents the results of these analyses, detailing the impacts of the surgical intervention on patient outcomes as measured by the specified methods.

## Results

**Fig. 2:**
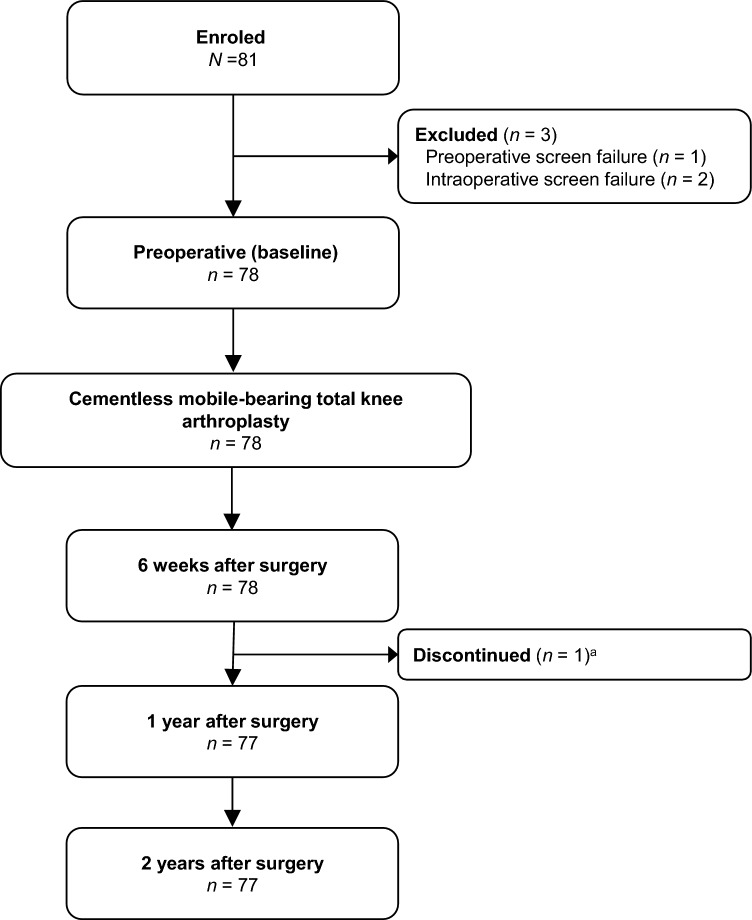
Flowchart of the patients in this study. ^a^One patient was withdrawn from the study 244 days after surgery owing to death from pancreatic carcinoma.

Seventy-eight patients (78 knees) underwent surgery with the newly introduced cementless mobile-bearing TKA system. Three patients were excluded from the study owing to pre- or intraoperative screening failure; one patient did not meet the age criteria, and two patients used cemented implant due to their condition. One patient was withdrawn from the study owing to death from pancreatic carcinoma. The event was independent from knee osteoarthritis and the event occurred 244 days after surgery, it was evaluated not related to the procedure or device (Fig. 2). A summary of the patients’ demographic data is shown in Table 1.

**Table 1:** Patients’ demographic and baseline characteristics, and intra- and perioperative variables.

	Total (*N* = 78)
Age (years), mean ± SD	70.9 ± 5.9
Female, *n* (%)	54 (69.2%)
BMI (kg/m^2^), mean ± SD	26.5 ± 4.0
Primary diagnosis Osteoarthritis, *n* (%)	78 (100.0%)
Surgical duration (skin to skin) (min), mean ± SD	71.9 ± 26.6
Patella resurfaced, *n* (%)	8 (10.3%)
Posterior cruciate ligament treatment, *n* (%)	
Resected	57 (73.1%)
Retained	21 (26.9%)

*BMI* body mass index, *SD* standard deviation.

### Clinical Evaluations

Among the 78 patients, the means of all subscores for the KOOS, PKIP, 2011KSS, EQ-5D-3L and SKO improved over time and showed significant improvement (*p* < 0.05, two-sided paired t-test) at 6 weeks, 1 year, and 2 years after surgery compared with preoperative baseline values (Table 2). The mean (± SD) change in the KOOS-Activities of Daily Living subscore from baseline to 2 years after surgery was 25.3 ± 15.2 (95% CI 21.9–28.8); similar trends were found for the other subscores.

**Table 2:** Clinical outcomes evaluated using PROMs.

	Preoperative baseline *n* = 78	After surgery		
		6 weeks *n* = 78	1 year *n* = 77	2 years *n* = 77
KOOS				
ADL	58.0 ± 16.2	70.6 ± 13.7	82.6 ± 13.3	83.6 ± 13.8
Pain	44.4 ± 15.8	62.0 ± 15.8	81.6 ± 11.9	84.3 ± 13.5
Symptoms	50.7 ± 18.6	62.5 ± 13.6	78.8 ± 10.9	81.6 ± 11.5
Sports/recreation	22.4 ± 18.4	27.3 ± 22.2	49.6 ± 24.3	54.2 ± 25.4
QOL	26.1 ± 16.3	46.1 ± 20.2	63.9 ± 20.6	65.4 ± 25.0
PKIP				
Total	26.3 ± 13.8	42.9 ± 12.1	54.6 ± 15.4	58.9 ± 18.6
Confidence	3.1 ± 2.0	4.6 ± 1.6	5.7 ± 2.1	6.0 ± 2.3
Modification of activities	4.2 ± 3.0	5.8 ± 2.3	5.9 ± 3.1	6.4 ± 2.7
Satisfaction	2.2 ± 1.3	4.5 ± 1.6	6.0 ± 1.8	6.4 ± 2.0
Stability	2.5 ± 1.7	4.4 ± 1.7	5.8 ± 2.1	6.1 ± 2.3
Knee awareness	1.2 ± 1.8	2.2 ± 2.1	3.9 ± 2.9	4.6 ± 3.1
2011KSS				
Total	32.7 ± 19.2	82.5 ± 7.6	90.4 ± 8.5	91.5 ± 11.0
Symptoms	8.1 ± 5.0	15.8 ± 4.2	19.8 ± 4.1	20.0 ± 5.4
Satisfaction	13.6 ± 5.2	19.3 ± 6.1	24.7 ± 6.8	26.7 ± 8.2
Expectation	13.9 ± 1.4	8.6 ± 2.6	9.2 ± 2.2	9.6 ± 3.1
Functional activity	47.9 ± 18.2	58.9 ± 16.6	76.0 ± 17.0	74.9 ± 17.3
EQ-5D-3L				
Index	0.6 ± 0.2	0.7 ± 0.1	0.9 ± 0.2	0.9 ± 0.2
VAS	61.5 ± 17.0	69.4 ± 17.4	79.0 ± 14.0	81.1 ± 15.0
SKO				
Pain at rest	4.9 ± 3.1	3.7 ± 2.3	1.1 ± 1.6	1.2 ± 1.8
Pain during activity	6.2 ± 2.8	4.1 ± 2.1	1.6 ± 1.7	1.6 ± 2.0

All subscores are shown as mean ± standard deviation.

Subscale scores showed significant improvement at 6 weeks, 1 year, and 2 years after surgery compared with preoperative baseline values using a two-sided paired t-test (*p* < 0.05).

*ADL* activities of daily living, *EQ-5D-3L* European Quality of Life 5 Dimensions—3 Levels, *KOOS* Knee Injury and Osteoarthritis Outcomes Score, *2011KSS* 2011 Knee Society Score, *PKIP* Patient’s Knee Implant Performance, *PROM* patient-reported outcome measure, *QOL* quality of life, *SKO* Subject Knee Outcomes, *VAS* visual analogue scale.

### Radiographic evaluations

The mean (± SD) distal femoral valgus angle (α), proximal tibial varus angle (β), femoral flexion angle (γ) and tibial slope (σ) (Fig. 1) at 2 years after surgery were 95.8° ± 2.3, 90.3° ± 1.9, 4.5° ± 3.3 and 89.5° ± 3.8, respectively. The mean (± SD) FTA at baseline was 182.9° ± 5.6 and improved to 174.3° ± 3.4 at 2 years after surgery. The mean (± SD) range of motion knee extension was 0.3° ± 2.3 at 2 years after surgery, which indicated significant improvement (*p* < 0.001, two-sided paired t-test) compared with the baseline value of 8.3° ± 7.2. The mean (± SD) range of motion knee flexion at baseline was 125.4° ± 10.3 and 123.7° ± 10.7 at 2 years after surgery which did not show significant difference using two-sided paired t-test.

**Fig. 3:**
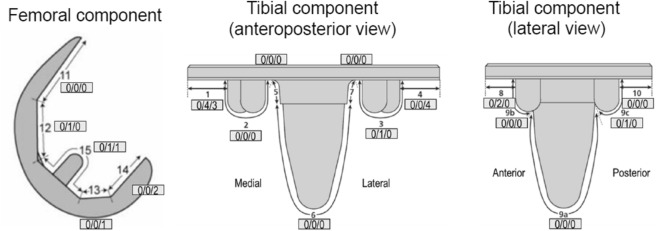
RLLs by zone on the femoral and tibial components. The figure shows the numbers of patients with an RLL of ≥ 1 mm in each zone 6 weeks, 1 year, and 2 years after surgery. Two patients with RLLs in different zones were counted as *n* = 1 in each zone. *RLL* radiolucent line.

**Fig. 4:**
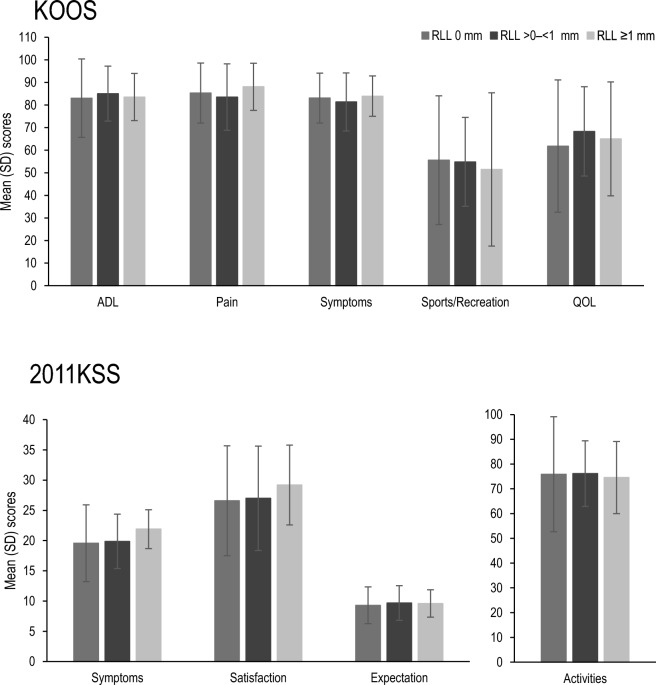
PROMs evaluated by KOOS and 2011KSS scores 2 years after surgery by RLL width. No significant difference was found in any of the KOOS and 2011KSS subscores by RLL width (0 mm, > 0 to < 1 mm and ≥ 1 mm). *ADL* activities of daily living, *KOOS* Knee injury and Osteoarthritis Outcomes Score, *2011KSS* 2011 Knee Society Score, *PROM* patient-reported outcome measure, *QOL* quality of life, *RLL* radiolucent line, *SD* standard deviation.

No RLL was detected for any component 6 weeks after surgery (Table 3). RLLs of ≥ 1 mm were seen at the femoral component interface in 2.8% and 5.7% of the patients 1 and 2 years after surgery, respectively. RLLs ≥ 1 mm underneath the tibial tray were seen in 9.7% and 10.9% of the patients 1 and 2 years after surgery, respectively. One and 2 years after surgery, a higher number of patients had RLLs in zones 1, 4 and 8 (Fig. 3) compared with the number of patients with RLLs in the other zones. Only one patient had an RLL ≥ 2 mm for the tibial component 1 year after surgery. Other signs, such as osteolysis, aseptic loosening, septic loosening, bone fracture, subsidence and migration were not detected. There were also no trends between the KOOS and 2011KSS subscores after analysis, regardless of the presence of RLLs (Fig. 4).

**Table 3:** RLLs on the femoral/tibial components 6 weeks, 1 year, and 2 years after surgery.

	6 weeks	1 year	2 years
Femur			
RLL ≥ 1 to < 2 mm	0/72 (0%)	2/71 (2.8%)	4/70 (5.7%)
RLL ≥ 2 mm	0/72 (0%)	0/71 (0%)	0/70 (0%)
Tibia			
RLL ≥ 1 to < 2 mm	0/44 (0%)	5/62 (8.1%)	7/64 (10.9%)
RLL ≥ 2 mm	0/44 (0%)	1/62 (1.6%)	0/64 (0%)

Values are shown as *n/N* (%). *n* indicates the number of patients with the finding, and *N* indicates the number of patients with no missing values for the measurement. A patient with a finding was counted only once for the finding. Six patients at 6 weeks and 1 year, respectively, and seven patients 2 years after surgery were not evaluable owing to poor quality of the radiographic images of the femur. Thirty-four patients at 6 weeks, 15 patients at 1 year and 13 patients at 2 years after surgery were unevaluable owing to the poor quality of the radiographic images of the tibia.

*RLL* radiolucent line.

Uni- and multivariate analyses showed that six of the seven investigated factors (age, sex, BMI, range of motion (flexion), posterior cruciate ligament treatment and β angle) were not correlated with the occurrence of all RLLs. However, an FTA of < 173° was associated with a lower incidence of all RLLs compared with FTAS of ≥ 173° and < 176° (Table 4).

**Table 4:** Logistic regression analysis of all RLLs 2 years after surgery.

Variables	Univariate analysis of covariates for all RLLs 2 years after surgery			Multivariate analysis of covariates for all RLLs 2 years after surgery		
	No. of patients in the analysis	OR and 95% CI^a^	*p*-value	No. of patients in the analysis	OR and 95% CI^a^	*p*-value
Age (years) (ref: ≤ 70)	64	0.79 (0.28–2.19)	0.65	64	0.69 (0.21–2.27)	0.54
Male (ref: female)	64	1.86 (0.62–5.54)	0.27	64	1.39 (0.35–5.49)	0.64
BMI (kg/m^2^) ≥ 25 (ref: < 25)	64	1.22 (0.45–3.30)	0.70	64	1.10 (0.35–3.44)	0.87
Range of motion, knee flexion (degrees) ≥ 120 (ref: < 120)	64	3.39 (0.79–14.57)	0.10	64	4.05 (0.76–21.67)	0.10
Posterior cruciate ligament (ref: intact + balanced/partial release)	64	0.80 (0.21–3.09)	0.75	64	0.54 (0.11–2.55)	0.44
FTA (degrees) < 173 (ref: ≥ 173 to < 176)	64	0.25 (0.07–0.94)	0.04	64	0.16 (0.03–0.74)	0.02
FTA (degrees) ≥ 176 (ref: ≥ 173 to < 176)	64	0.83 (0.26–2.72)	0.37	64	0.84 (0.21–3.38)	0.28
β angle (degrees) < 87 or > 93 (ref: 87–93)	64	1.74 (0.30–10.27)	0.54	64	4.02 (0.43–37.80)	0.22

Uni- and multivariate logistic regression models were used to estimate the unadjusted and adjusted odds ratios, respectively.

^a^The parameter estimate was based on modelling for the RLL; therefore, an OR of > 1 indicates a greater likelihood of an RLL.

*BMI* body mass index, *CI* confidence interval, *FTA* femorotibial angle, *OR* odds ratio, *RLL* radiolucent line.

### Complications

One patient developed a procedure-related staphylococcal infection 5 days after the surgery, which resolved with debridement and antibiotic therapy without implant revision. There were no device-related adverse events, and no patients required device revision or reoperation during the study period.

## Discussion

This study found no significant difference in PROMs between patients with and without RLLs 2 years following implantation with the newly introduced cementless mobile-bearing TKA system. The results suggest that the existence of RLLs may not affect short-term clinical outcomes. RLLs were seen at both femoral and tibial components, however, fortunately there was no RLL with entire circumference during the study. As the RLLs were detected just partially, it was considered that the implant fixation is stable and those RLLs did not impact PROMs. These findings should help reduce the hesitation of surgeons to select cementless fixation, owing to concerns regarding RLLs in the early clinical stage. According to the data for all patients, the PROMs improved and pain subsided as early as 6 weeks after surgery, and these results were sustained for up to 2 years after surgery. The mean change in the KOOS-Activities of Daily Living subscore from baseline to 2 years after surgery was comparable to the reported minimal clinically important improvement after TKA^17,18^.

On the basis of the radiographic evaluations, this study demonstrated that the incidence of RLLs with the newly introduced cementless mobile-bearing TKA system 1 year after surgery was 2.8% in the femur and 9.7% in the tibia. These values were lower than those in a report of a former design of a cementless mobile-bearing TKA system (LCS) 1 year after surgery, at 4% in the femur and 43% in the tibia^15^. These data suggest that the design of the new tibial base, which was designed to encourage biological fixation, effectively reduced RLLs. The former system has provided excellent functional results and wear rates in long-term follow-up analyses^19,20^. Regarding the incidence of RLLs by zone, more RLLs were found in the posterior condyle of the femur and medial and lateral tibia compared with the number of RLLs found in other locations, similar to the findings for the former design^21^. Accumulating evidence suggests that the incidence of RLLs tends to increase 1–2 years after surgery and gradually decreases after 5–10 years^15^. These findings are supported by several reports that demonstrated a temporary increase in the incidence of RLLs after TKA^11,19,21^. Although aseptic loosening is one of the most common causes of failure after TKA^22^, subsequent revision owing to loosening have not been reported. The results of this study suggest that the newly introduced cementless mobile-bearing TKA system provides short-term fixation without any loosening.

According to the National Joint Registry Annual Report 2023, cumulative revision rate of the same design implant for cemented version at 5 years and 10 years post primary TKA were 1.33% and 2.01%, respectively^23^. Comparing to this data in the same annual report, cumulative revision rate of all cemented implants at 5 years and 10 years post primary TKA were 2.04% and 3.11%. respectively. The revision rate of Attune mobile-bearing cemented version was considered comparable with the other cemented implants.

Recent reviews showed the comparable functional outcomes and survivorship between cemented and cementless TKA^24,25^. However, concerns remain regarding early failure of cementless fixation in patients with factors that potentially increase the risk of loosening. To investigate the risk factors for the occurrence of RLLs, uni- and multivariate analyses were performed for seven factors: age, sex, BMI, range of motion, posterior cruciate ligament treatment, FTA and β angle. The results showed no correlation between six of the factors and the presence of RLLs; however, the incidence of RLLs was lower in patients with an FTA of < 173° compared with that in patients with FTAs ≥ 173° and < 176°. Postoperative alignment may influence the load distribution to the periprosthetic bone and may affect the occurrence of RLLs 2 years after surgery. Although the incidence of RLLs was not correlated with PROMs in this study, longer-term follow-up of RLLs is necessary as future research.

One patient developed post operative infection, which was evaluated not related to the study device. As the event resolved with debridement and antibiotic therapy without implant revision, it was considered that this event do not affect the study assessments.

Overall, this study demonstrated that the newly introduced cementless mobile-bearing TKA system significantly improved knee functional outcomes and patient satisfaction, including pain, without raising new safety concerns in Japanese patients with osteoarthritis for up to 2 years after surgery. Additionally, the results of the multivariate analysis suggest that this TKA system could be reliable for various patients. Cementless fixation is preferred by some surgeons in the treatment of younger patients because the procedure preserves bone stock compared with cemented fixation designs, and the fixation achieved is physiological and can respond to stresses physiologically^9,26^. The results of this study suggest that cementless fixation can also be performed effectively and safely in older patients.

This study has limitations, the first of which is the single-arm design without consecutive enrolment. The study results showed a lower incidence of RLLs compared with a previous report of a former design^15^, and PROMs improved over time after surgery. However, randomized comparative study adjusting patient background is required to clarify these points. The second limitation is the short follow-up period. A previous report showed that the incidence of RLLs with a cementless TKA increased 1–2 years after surgery and gradually decreased after 5–10 years^15^. Therefore, a 2-year follow-up for RLLs is adequate for early feedback on the newly introduced cementless TKA. As the report for the former design examined data up to 10 years after surgery, it is necessary to confirm the long-term results of the newly introduced system used in this study as well to confirm the biological fixation of the implant. The third limitation is the small sample size and the loss of follow-up imaging for several patients, owing to poor radiographic image quality. The sample size of this study was determined using the KOOS subscore to confirm the short-term performance of the implant during clinical use in the Japanese patients at a limited number of sites specialized in TKA, before introducing this implant widely in Japan. Comparative study with larger numbers of patients would be expected for obtaining more reliable data. While the findings are promising, further research is needed to assess long-term outcomes beyond the 2-year follow-up and in more diverse patient populations.

## Conclusion

This is the first report of the results of prospective multicentre study involving Japanese patients with osteoarthritis for the newly introduced cementless mobile-bearing TKA system. This newly introduced system demonstrated significant improvements in both knee function and pain reduction, as measured by KOOS, PKIP, 2011KSS, EQ-5D-3L and SKO, over a 2-year postoperative period without implant-related complications, such as loosening or new safety concerns in the early clinical stage. The lack of correlation between the incidence of RLLs and PROMs suggests that the presence of these radiographic changes does not impact the patient’s perceived outcome or quality of life in the short-term follow-up period. These findings support the broader adoption of the cementless mobile-bearing TKA system in similar patient populations, as they indicate a reliable postoperative outcome without increased risk of complications.

## Data Availability

All data generated or analysed during this study are included in this published article.
